# Transcriptional expression of m^6^A and m^5^C RNA methyltransferase genes in the brain and fat body of honey bee adult workers

**DOI:** 10.3389/fcell.2022.921503

**Published:** 2022-08-29

**Authors:** Luana Bataglia, Zilá Luz Paulino Simões, Francis Morais Franco Nunes

**Affiliations:** ^1^ Departamento de Genética, Faculdade de Medicina de Ribeirão Preto, Universidade de São Paulo, Ribeirão Preto, Brazil; ^2^ Departamento de Biologia, Faculdade de Filosofia Ciências e Letras de Ribeirão Preto, Universidade de São Paulo, Ribeirão Preto, Brazil; ^3^ Departamento de Genética e Evolução, Centro de Ciências Biológicas e da Saúde, Universidade Federal de São Carlos, São Carlos, Brazil

**Keywords:** RNA methylation, m^6^A, m^5^C, epitranscriptomics, bee, nutrition, aging, behavior

## Abstract

Honey bee (*Apis mellifera*) adult workers change behaviors and nutrition according to age progression. Young workers, such as nurses, perform in-hive tasks and consume protein-rich pollen, while older workers (foragers) leave the colony to search for food, and consume carbohydrate-rich nectar. These environmentally stimulated events involve transcriptional and DNA epigenetic marks alterations in worker tissues. However, post-transcriptional RNA modifications (epitranscriptomics) are still poorly explored in bees. We investigated the transcriptional profiles of m^6^A and m^5^C RNA methyltransferases in the brain and fat body of adult workers of 1) different ages and performing different tasks [nurses of 8 days-old (N-8D) and foragers of 29 days-old (F-29D), sampled from wild-type colonies], and 2) same-aged young workers caged in an incubator and treated with a pollen-rich [PR] or a pollen-deprived [PD] diet for 8 days. In the brain, METTL3, DNMT2, NOP2, NSUN2, NSUN5, and NSUN7 genes increased expression during adulthood (from N-8D to F-29D), while the opposite pattern was observed in the fat body for METTL3, DNMT2, and NSUN2 genes. Regarding diet treatments, high expression levels were observed in the brains of the pollen-deprived group (DNMT2, NOP2, and NSUN2 genes) and the fat bodies of the pollen-rich group (NOP2, NSUN4, and NSUN5 genes) compared to the brains of the PR group and the fat bodies of the PD group, respectively. Our data indicate that RNA epigenetics may be an important regulatory layer in the development of adult workers, presenting tissue-specific signatures of RNA methyltransferases expression in response to age, behavior, and diet content.

## Introduction

The adult life of honey bee (*Apis mellifera*) workers is characterized by a temporal polyethism, in which individuals progressively experience different tasks and behaviors during their lifetime. This age-related polyethism is accompanied by alteration of the feeding regimes. Young workers feed on pollen, a predominant source of protein, lipids, vitamins, and minerals (Brodschneider and Crailsheim, 2010). During this period, they perform in-hive tasks, such as feeding the developing larvae, a behavior that characterizes them as nurses (Winston, 1987; Page and Peng, 2001). Around the third week of adulthood, workers start to go outside the colony to collect pollen, nectar, resins, and water, a behavior that characterizes them as foragers (Winston, 1987; Page and Peng, 2001). The foragers consume nectar, a diet rich in carbohydrates and low in proteins, which increases the bee’s energy metabolism, necessary for flight activity and resource gathering (Winston, 1987). This behavioral transition in honey bees involves transcriptional and metabolic changes in tissues, especially in the brain and the fat body (Ament et al., 2011). Foragers have an increase in cognitive capacity (memory and learning) compared to young workers performing in-hive tasks (Zayed and Robinson, 2012), and their brain transcriptomes differ significantly (Whitfield et al., 2003). The fat body is a metabolic center and during the behavior transition, the foragers lose half of their lipid stores and the capacity to metabolize these molecules (Toth and Robinson, 2005).

Epigenetics is proposed to be a plastic mechanism for regulating gene expression in response to environmental conditions (Vaiserman, 2014; Abdul et al., 2017). Diet is one of the external factors that alters gene expression through epigenetic modifications of the genomic DNA (epigenomics). The nutrients and bioactive components present in food can alter the activity of genes involved in DNA methylation or histone modifications or can change the availability of substrates required for these modifications’ reactions (reviewed by Abdul et al., 2017). In honey bees, the different diets consumed by workers and queens’ larvae trigger distinct abundance and patterns of DNA methylation between castes (Kucharski et al., 2008). In workers, several DNA CpG sites are differentially methylated comparing the brains of nurses and foragers (Herb et al., 2012; Lockett et al., 2012). Although knowledge in bee epigenomics has advanced in recent years, little has been explored about how epitranscriptomics, an emerging field of investigation, acts in bee development.

Epitranscriptomics is the study of RNA post-transcriptional modifications. More than 170 chemical modifications present in RNAs are currently known (Boccaletto et al., 2017; Huang et al., 2020). Two of them, the methylation of the nitrogen N6 of adenosine (N6-methyladenosine, m^6^A) and the methylation of the carbon C5 of cytosine (5-methylcytosine, m^5^C) are the most studied and regulate several biological processes such as development in mouse (Tuorto et al., 2012) and *Drosophila melanogaster* (Lence et al., 2016), behavioral adaptation in mammals (Meyer et al., 2012), aging and response to environmental (feeding, temperature) stimuli in *D. melanogaster*, *Caenorhabditis elegans* and *Saccharomyces cerevisiae* (Schosserer et al., 2015). Interestingly, these biological processes are closely related to those observed in the context of age polyethism such that RNA modifications have become excellent candidates to be explored in honey bees.

To date, over 1,000 RNA epigenetic studies were published, however, only 3% of them investigated insects (source: PubMed, June 2022). Most of them used *D. melanogaster*, with RNA methylation being related to sex determination (Haussmann, et al., 2016; Kan et al., 2017), fertility (Hongay and Orr-Weaver, 2011), and nervous system development (Lence et al., 2016). In *Bombyx mori*, m^6^A was reported to control gene expression and cell cycle progression (Li et al., 2019). In honey bees, two studies revealed RNA modifications acting in caste differentiation (Bataglia et al., 2021; Wang et al., 2021). Our group recently identified the orthologs of RNA methyltransferase genes in the *A. mellifera* genome, being METTL3 (methyltransferase like 3) and METTL14 (methyltransferase like 14) for m^6^A methylation, and DNMT2 (DNA methyltransferase 2) and the NSUN (NOP2/Sun RNA methyltransferase member) family encoding genes (NOP2, NSUN2, NSUN4, NSUN5, and NSUN7) for m^5^C methylation (Bataglia et al., 2021).

Here, we verified if the expression of m^6^A and m^5^C RNA methyltransferase genes vary in the development of *A. mellifera* adult workers according to age-related behavior (nurses of 8 days-old versus foragers of 29 days-old), diet content (pollen-rich or pollen-deprived), or tissue (brain or fat body). We found that the expression of some RNA methyltransferase transcripts is modulated during temporal polyethism in a tissue-specific and nutrition-dependent manner.

## Materials and methods

### Samples

*Apis mellifera* workers were collected from three queenright colonies maintained at the experimental apiary of the Universidade de São Paulo in Ribeirão Preto, Brazil. Three independent biological experiments were performed, each one starting with 1 day of difference, on three consecutive days, each day with bees from a different colony. For each experiment, combs containing pharate adults close to emergence (Pbd stage) were placed in an incubator at 34°C and ∼80% relative humidity for 8 h to obtain at least 400 newly-emerged adult workers.

Each experiment was performed according to the following design. A total of 100 newly-emerged workers were divided (day 1) into two cages (*n* = 50 per cage of 20 cm × 13 cm × 13 cm) and kept in the incubator, with food and water *ad libitum* for 8 days. Every day, the food and the water were replaced, and the dead bees were removed. The bees of one cage received a pollen-deprived (PD) diet (a mix of 20% honey plus 80% powdered sugar, named candy), and the bees of the other cage received a pollen-rich (PR) diet (a mix of 70% candy and 30% of fresh poly floral pollen collected from the same apiary). On day 8, five individuals per cage were collected. The newly-emerged workers that were not placed in cages (at least 300 workers), were then marked on the thorax with uni POSCA non-toxic pens (1.8–2.5 mm), for age control, and returned to their original colony. We collected five nurses of 8 days-old visiting larval cells and five foragers of 29 days-old returning to the colony (N-8D and F-29D, respectively).

The collected workers were anesthetized at 4°C for 5 min for tissue dissection. Then, we quickly dissected the brain and the fat body of each worker. Brains were dissected using a Leica M125 stereomicroscope and all glands and adjacent tissues were removed. For sampling the fat body, we pulled the sting along with the last abdominal segment, to remove the venom gland, the intestine, and adjacent tissues (such as the ovaries), leaving only the abdominal carcass. The abdominal carcass is internally lined by the fat body (Ament et al., 2011). Each individual brain or abdominal fat body was placed separately in tubes containing TRIzol^®^ (Invitrogen) for RNA extraction.

### Total RNA extraction, cDNA synthesis, and real-time PCR

The total RNA was extracted from individual brains and fat bodies according to Trizol^®^ protocol. To avoid genomic DNA contamination, the total RNA was treated with DNase (DNase I Amplification Grade—Invitrogen). Next, cDNA was synthesized by reverse transcription catalyzed by SuperScript II reverse transcriptase (200 U/μL, Invitrogen) and oligo (dT)12–18 primers.

Real-time PCR was performed with 60 brain samples and 60 fat body samples. Among these 60 samples of each tissue, 15 samples represent each condition (PD, PR, N-8D, F-29D), being five samples of each experiment. The expression of the following methyltransferase genes was evaluated: for m^6^A, METTL3 (NCBI Gene ID: 551911) and METTL14 (409900), and for m^5^C, DNMT2 (410512), NOP2 (726212), NSUN2 (411579), NSUN4 (102655259), NSUN5 (410262), and NSUN7 (413907).

We perform the real-time PCR following the PowerUP™ SYBR™ Green Master Mix (2X) protocol and cycling (Applied Biosystems). For each sample, reactions were assembled in triplicate, and every single reaction consisted of a final volume of 20 μl, containing: 10 μl of PowerUP™ SYBR™ Green Master Mix (2X) solution (Applied Biosystems), 6.4 μl of water, 0.8 μl of each primer, forward and reverse (10 pmol/μL), and 2 μl of the cDNA solution (10 ng/μl). The primer pairs used are listed in Supplementary Table S1, including the pair used for amplification of the ribosomal protein L32 (RpL32) encoding gene, used as a reference for data normalization (Grozinger et al., 2003; Lourenço et al., 2008). The amplification reactions were conducted on a 7,500 Real-Time PCR System (Applied Biosystems). For primer pairs with optimal efficiency at 60°C, the following cycling conditions were used: 50°C for 2 min, 95°C for 10 min, followed by 40 cycles of 95°C for 15 s and 60°C for 1 min. For primer pairs with annealing temperature lower than 60°C, the following cycle was used: 50°C for 2 min, 95°C for 10 min, followed by 40 cycles of 95°C for 15 s, primer annealing temperature for 15 s, and 72°C for 1 min.

The relative expressions were calculated using the 2^-∆∆CT^ method (Livak and Schmittgen, 2001). The statistical analyses were performed in R (version 4.1.3). We applied the Generalized Linear Mixed (GLM) models (package lme4 version 1.1–29) followed by the Anova function (package car version 3.0.12) to compare the different diets or ages with gene expression, using colony or cage replicate as a random effect. Significant outliers were removed [Grubbs’ test (alpha = 0.05)] when detected.

## Results

**FIGURE 1:**
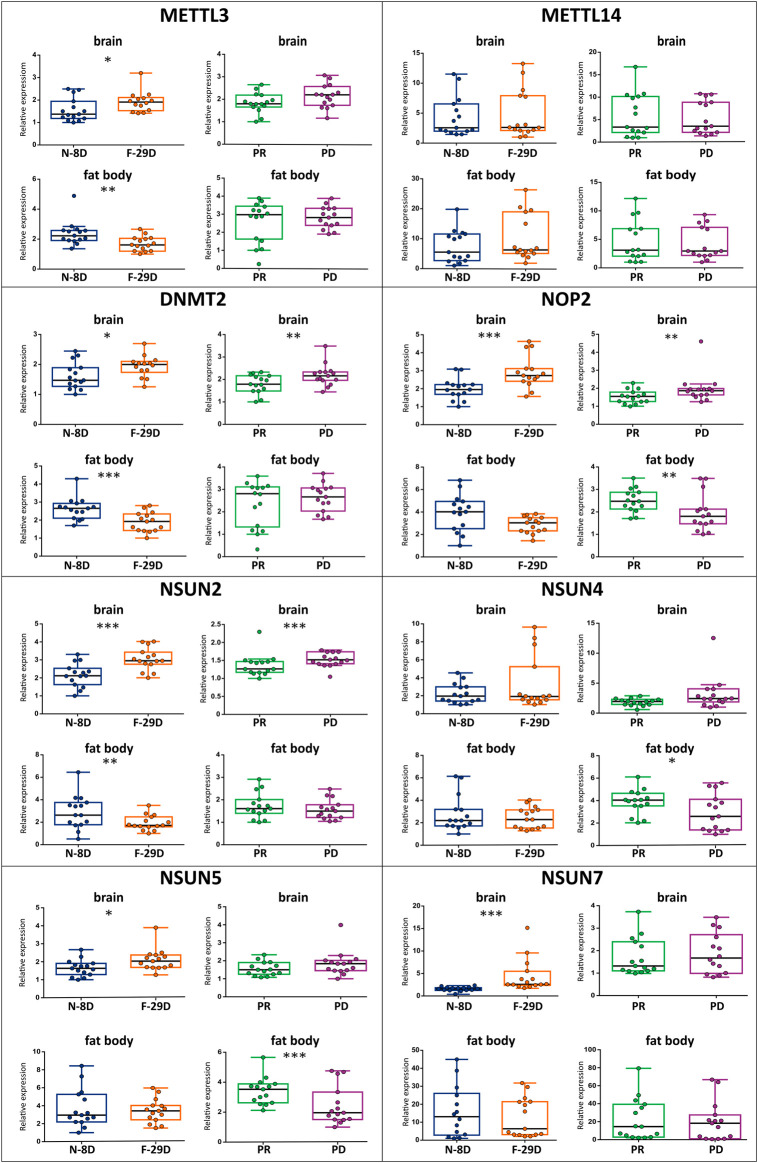
Relative expression of m^6^A (METTL3 and METTL14) and m^5^C (DNMT2, NOP2, NSUN2, NSUN4, NSUN5, and NSUN7) RNA methyltransferase genes, measured by real-time PCR in the brain and fat body samples from workers of different ages/behaviors (N-8D for 8 days-old nurses–blue—and F-29D for 29 days-old foragers–orange) and under different dietary regimes (PR for pollen-rich diet–green—and PD for pollen-deprived–purple), *n* = 15 per condition. The boxplots represent the minimum, maximum, and median values. The presence of asterisks indicates a significant difference in expression, *p < 0.05, *p < 0.01, **p < 0.001.

The expression of METTL3, DNMT2, NOP2, NSUN2, NSUN5, and NSUN7 methyltransferases statistically vary in the brain of workers of different ages, performing different tasks (N-8D versus F-29D comparison). Also, DNMT2, NOP2, and NSUN2 were differentially expressed in the brain of same-aged young workers that consumed distinct diets (PR versus PD comparison). In the fat body, the expression of METTL3, DNMT2, and NSUN2 genes were influenced by age/task (N-8D versus F-29D comparison), while the expression of NOP2, NSUN4, and NSUN5 was influenced by diet (PR versus PD comparison). All these results are graphically represented in Figure 1 (and the results of the statistical analyses are in Supplementary Table S2).

A clear expression pattern for methyltransferase genes should be highlighted. Regarding the gene set that presented significant transcriptional differences in the brain, we observed lower levels in both N-8D and PR groups when compared to F-29D and PD groups. In addition, an opposite pattern was observed in the fat body, with increased expression in N-8D and PR groups in relation to F-29D and PD groups.

## Discussion

The adult life progression of *A. mellifera* workers is marked by changes ranging from behavior to nutrition, mainly in the brain and the fat body (Ament et al., 2011). Despite the age-related division of labor in workers (temporal polyethism) being well-documented (Page et al., 2006), the participation of RNA modifications in these processes remains largely unknown (Bataglia et al., 2021). To advance this knowledge, we have investigated the transcriptional expression of m^6^A and m^5^C RNA methyltransferases in the brain and fat body of workers of different ages, performing different tasks, or same-aged young workers that consumed a pollen-deprived or a pollen-rich diet.

Foraging tasks of workers require well-developed brain abilities such as, recognizing the environment, identifying food resources, and communicating this information with nestmates. During the transition from in-hive to outside tasks, structural modifications such as the increase of the mushroom bodies (Withers et al., 1993; Durst et al., 1994) and the neuronal dendritic arborization (Farris et al., 2001) are observed. Higher expression levels of METTL3, DNMT2, NOP2, NSUN2, NSUN5, and NSUN7 transcripts were found in the brain of foragers compared to nurses. All these genes are reported in the literature as being involved in brain development, memory formation, or increase of cognitive capacity. The overexpression of METTL3 enhances hippocampus-dependent long-term memory in mice (Zhang et al., 2018) while its depletion perturbs short-term memory formation in flies (Kan et al., 2021). High abundance of mRNAs of DNMT2 (Rai et al., 2007) and NOP2 (Kosi et al., 2015) are related to neurogenesis in zebrafish brains and neural stem cell proliferation in mammals, respectively. The lack of NSUN2 expression inhibits normal brain development by reducing the number of differentiated neurons in mice (Flores et al., 2017) and short-term memory deficits in *Drosophila* (Abbasi-Moheb et al., 2012). NSUN5 mutant mice showed deficits in spatial cognitive abilities (Zhang et al., 2019) and in cerebral cortex development (Chen et al., 2019). Transcriptomic evidence suggests that NSUN7 may act on the cognitive processes of mammals (Chi and Delgado-Olguin. 2013; Tang et al., 2019). It is increasingly evident that the development of the nervous system, cognitive and behavioral aspects, and learning and memory abilities, in different organisms, are associated with a reprogramming (spatial and temporal) of the epitranscriptome (Jung and Goldman, 2018; Leighton et al., 2018). Our study supports and extends such evidence.

The main function of fat body of insects is the metabolism of macromolecules (reviewed by Skowronek et al., 2021). RNA methylation acts on nutritional metabolism, mainly on glucose and lipid metabolism (Wu et al., 2020). For example, METTL3 knockdown decreased lipid accumulation in human liver cell culture (Zhong et al., 2018). The difference in the expression of RNA methyltransferases in the fat body of nurses and foragers found here may be integrated into the gene regulatory network associated with the conversion of a protein-lipid metabolism in younger workers to a carbohydrate metabolism in older ones (Ament et al., 2011). Our data showed that not only age, but diet also influenced the expression of some methyltransferases. NOP2, NSUN4, and NSUN5 were differentially expressed in the fat body of same-aged workers that fed on different diets under caged conditions and did not perform behavioral tasks. Thus, these genes are excellent candidates for further studies about nutritional metabolism uncoupled to social interactions in honey bees. Moreover, in the worker brains, DNMT2, NOP2, and NSUN2 expression levels were diet-responsive. Our data are further corroborated by a study performed with rats fed on high or low-calorie diets, that showed the nutritional influence on the differential expression of RNA methyltransferases depending on the tissue analyzed (D’Aquila et al., 2022). It is important to highlight that the pollen-rich (PR) diet mimics that consumed by nurses while the pollen-deprived (PD) diet is similar to the nutritional content of foragers. Recently, Martelli et al. (2022) showed that young honey bee workers fed on PD diet age precociously and present biological parameters similar to foragers. Here, we observed corresponding expression profiles of some m^5^C genes (DNMT2, NOP2, and NSUN2) between N-8D + PR groups, which contrast with data from F-29D + PD groups. These genes will be tested in the future as potential candidates for aging markers in adult worker brains.

The fat body is also important for the production of energy and for the synthesis of immune system proteins (reviewed by Skowronek et al., 2021). Changes in the activity of methyltransferases have already been observed in response to stressful conditions. METTL3 plays a crucial role in oxidative stress, glycolytic stress, and DNA damage, and promotes an increased lipid metabolism in different mammalian cell lines (reviewed by Wilkinson et al., 2021). DNMT2 integrates stress response pathways by protecting tRNAs against ribonuclease cleavage induced by heat shock conditions in *Drosophila* (Schaefer et al., 2010), while NOP2 is involved with resistance to heat stress in *C. elegans* (Heissenberger et al., 2020). In mice, NSUN2 is required in cellular adaptation in response to toxic compounds and UV radiation (Gkatza et al., 2019). In honey bees, nurses have a more robust immune response to stress conditions compared to foragers (Amdam et al., 2004; Lourenço et al., 2019). In addition, our findings on the higher expression of some methyltransferases in the fat body of young bees compared to old ones seem somewhat coherent with this immunological scenario. Further research is needed to check if this suggestive crosstalk between immunity and epitranscriptomics is also true for bees.

This scientific topic is promising and some studies already involve epigenetic mechanisms to the xenobiotic response in insects. Epigenomic marks are associated with modulation of the expression of pesticide resistant genes in the beetle *Meligethes aeneus* (Erban et al., 2017). Also, strong evidence suggests that the expression of METTL3, METTL14 and a set of detoxification P450 genes along with increased levels of m^6^A marks confer thiamethoxam resistance to a strain of whitefly crop pest, *Bemisia tabaci* (Yang et al., 2021). It is important to consider that pollen is a common source of pesticides in agricultural sites and the contamination can be the cause of poisoning incidents of honey bees (Kiljanek et al., 2016). Although we did not screen the pollen for pesticides, our apiary is located within a non-agricultural area of a University with six million m^2^ containing an abundance of flowering plants representing nearly 300 species, and no poisoning effects were observed on bees we used as a source for the experiments. Furthermore, it is known that pollen consumption reduces the toxicity of pesticides in *Apis mellifera* (Barascou et al., 2021) and that diets influence the expression of genes of the epitranscriptomic machinery (this study). Perspectives are open to investigate whether RNA modifications respond to the detrimental impacts of insecticides and/or whether they confer some resistance to these pollinators.

Here we observed co-expression of RNA methyltransferase genes in all tested contexts in worker bees irrespective of their nutrition or age. Does this co-expression indicate functional redundancy? Do RNA methyltransferases have specific or integrated actions? It is widely accepted that all RNA methyltransferases have the canonical function of methylating RNAs, and RNA modifications are found in viruses (Kennedy et al., 2017), prokaryotes (Deng et al., 2015), yeasts (Clancy et al., 2002), plants (Fray & Simpson 2015), invertebrates (Hongay & Orr-Weaver, 2011), and vertebrates (D’Aquila et al., 2022). Despite METTL3 gene silencing in human embryonic stem cells is not sufficient for a complete reduction of m^6^A methylation levels, this knockdown at least removes the methylation marks of a specific subset of pluripotency-related genes, influencing the process of cell differentiation (Batista et al., 2014). This suggests a co-working, but it is also true that an individual RNA methyltransferase may have preferential RNA species as targets. NSUN2 preferentially methylates mRNA, tRNAs, and mitochondrial tRNAs (Tuorto et al., 2012; Shinoda et al., 2019), whereas NSUN5 preferentially methylates rRNA (Schosserer et al., 2015). Also, m^6^A sites are found mainly in mRNAs and affect the physical interaction of RNA with proteins or regulatory factors, resulting in altered translation or changes in mRNA stability (Meyer et al., 2015). This methylation also participates in alternative splicing (Zhao et al., 2014) and miRNA processing (Alarcón et al., 2015) events. The m^5^C marks are more frequent in non-protein-coding RNAs (ncRNAs, mainly tRNAs, and rRNAs) and maintain the stability of their secondary structures for the correct translation events (Micura et al., 2001; Schaefer et al., 2010). We believe that new findings should emerge in the coming years to clarify both species-specific or conserved functional issues related to epitranscriptomics. This is the case of a recent article that studied the m^5^C methylation patterns of maternal mRNAs in six species (five vertebrates: zebrafish, *Xenopus laevis*, *Xenopus tropicalis*, mouse, human, and one invertebrate: *D. melanogaster*) spanning ∼800 million years of evolution. For all tested species, they found a massive methylation of maternal mRNAs occurring in early embryonic development, and the rates drop dramatically after the mater-zygotic transition. This data shows an extremely conserved pattern in animals. On the other hand, they also observed regulatory innovations, such as the gain of m^5^C sites in mRNAs during evolution (Liu et al., 2022).

Concerning the differences we found in the expression of m^6^A and m^5^C methyltransferases between honey bee tissues, we speculate that they may reflect in the regulation of tissue-specific splicing variants previously found in the brain and fat body (Kannan et al., 2019), and/or several differentially expressed coding-genes and miRNA from workers performing different behaviors (Whitfield et al., 2003; Behura and Whitfield, 2010).

Bataglia et al. (2021) reported that global levels of m^6^A and m^5^C methylation quantified in the total RNA from brain and fat body samples increases in age progression. In this study we observed an increase in the expression of RNA methyltransferases in brain which is in accordance with the overall increase in global m^6^A and m^5^C methylation levels (Bataglia et al., 2021). However, in the fat body, although the higher expression of RNA methyltransferases is found in younger bees, the global m^6^A and m^5^C methylation levels are lower in young bees when compared with older bees. This point may sound contradictory, but it is important to emphasize that epitranscriptomic machinery is not only composed of RNA methyltransferases (named as writers) but also of readers and demethylases (erasers), and these last two sets of enzymes act in the recognition and removal of methyl groups, respectively (Schaefer et al., 2017). So, when the global methylation levels are accessed, we are measuring the resulting action of the whole machinery (writers, readers, and erasers) on the analyzed transcriptome.

We conclude that the adult life progression of *A. mellifera* workers is accompanied by changes in the expression of m^6^A and m^5^C RNA methyltransferases. It is possible that the mRNA methylome is dynamically regulated all through the lifespan of workers. We found that brain and fat body influence the transcriptional levels of these methyltransferases in opposite ways. Further the transcript levels of some of these genes are impacted by the protein content of food consumed as well as by the age-related behavior performed. Our results point to a tissue-specific transcriptional signature of RNA methyltransferases during the progression of adulthood.

## Data Availability

The raw data supporting the conclusions of this article will be made available by the authors, without undue reservation.
