# Supplementary material for: Transcriptional expression of m6A and m5C RNA methyltransferase genes in the brain and fat body of honey bee adult workers
Source: Front Cell Dev Biol. 2022 Aug 29;10:921503. doi: 10.3389/fcell.2022.921503 (PMC9467440; doi:10.3389/fcell.2022.921503)
Supplement: Supplementary file 1 [file DataSheet1.docx]

**Supporting Information – Table S1 and Table S2**

Transcriptional expression of m^6^A and m^5^C RNA methyltransferase genes in the brain and fat body of honey bee adult workers

Authors: Luana Bataglia, Zilá L. P. Simões, Francis M. F. Nunes

Table S1: Sequences (F-forward and R-reverse), expected amplicon sizes, and annealing temperatures of the primer pairs used in real-time PCR.

| **Primer** | **Sequence 5’→ 3’** | **Amplicon size (bp)** | **Annealing temperature** |
| --- | --- | --- | --- |
| METTL3-F | GACGAATCCTTGGGTGACTGT | 103 | 60°C |
| METTL3-R | CCTTTGGTTGAGTTGTTGGTCC |  |  |
| METTL14-F | ATTTTGATCGAGCCCCCGTT | 146 | 60°C |
| METTL14-R | CTACTGCCGCACCAGAGAAA |  |  |
| DNMT2-F | TGGTGGAATGCATTATGCCCT | 157 | 59°C |
| DNMT2-R | TATCTCTTGTGCAGAAAGTGATTGA |  |  |
| NOP2-F | CGCGTCGTCGTGATTTAGC | 114 | 60°C |
| NOP2-R | CACCCATGGGAACTTGCGAT |  |  |
| NSUN2-F | CCACCAAAACCTGAGGATGC | 135 | 60°C |
| NSUN2-R | TGATGCACGTTCCCAAGGTA |  |  |
| NSUN4-F | TTCAATCTGAGATTTTAGCGA | 112 | 55ºC |
| NSUN4-R | GCTACTTGTACAACACCATC |  |  |
| NSUN5-F | ATGTGTGCAGCTCCTGGAAT | 147 | 60°C |
| NSUN5-R | AGTTTCGACACAAGAAGCGTT |  |  |
| NSUN7-F | CTGTTTCCGAGCTCGTTTGT | 141 | 60°C |
| NSUN7-R | GTCGAGGTGCAAGTGAATGC |  |  |
| RpL32-F | CGTCATATGTTGCCAACTGGT | 150 | 60°C |
| RpL32-R | TTGAGCACGTTCAACAATGG |  |  |

**Table S2:** Statistical analyses details for the expression of the RNA methyltransferase genes. The table presents χ2 (chi-square), df (degree of freedom), and p-values resulting from the Generalized Linear Mixed models. Significant differences (p < 0.05) are represented in bold.

|  | | brain | | | | | | fat body | | | | | |
| --- | --- | --- | --- | --- | --- | --- | --- | --- | --- | --- | --- | --- | --- |
|  |  | N-8D X F-29D | | | PR X PD | | | N-8D X F-29D | | | PR X PD | | |
|  |  | χ2 | df | p-value | χ2 | df | p-value | χ2 | df | p-value | χ2 | df | p-value |
| genes | METTL3 | 5.7635 | 1 | **0.01636** | 3.0766 | 1 | 0.07943 | 9.3554 | 1 | **0.002223** | 0.6674 | 1 | 0.4139 |
|  | METTL14 | 0.0063 | 1 | 0.9368 | 0.069 | 1 | 0.7928 | 3.5812 | 1 | 0.05844 | 0.0154 | 1 | 0.9012 |
|  | DNMT2 | 5.5619 | 1 | **0.01836** | 8.2655 | 1 | **0.00404** | 14.088 | 1 | **0.000174** | 1.1143 | 1 | 0.2912 |
|  | NOP2 | 12.746 | 1 | **0.00035** | 7.2559 | 1 | **0.00706** | 2.9154 | 1 | 0.08774 | 7.7429 | 1 | **0.00539** |
|  | NSUN2 | 15.205 | 1 | **9.645e-05** | 17.281 | 1 | **3.223e-05** | 8.5468 | 1 | **0.003461** | 0.7546 | 1 | 0.385 |
|  | NSUN4 | 0.8434 | 1 | 0.3584 | 3.664 | 1 | 0.0556 | 0.1873 | 1 | 0.6652 | 5.711 | 1 | **0.01686** |
|  | NSUN5 | 8.1585 | 1 | **0.04286** | 1.4831 | 1 | 0.2233 | 0 | 1 | 0.9958 | 10.888 | 1 | **0.00096** |
|  | NSUN7 | 23.294 | 1 | **1.39e-06** | 0.1682 | 1 | 0.6817 | 0.0532 | 1 | 0.8176 | 0.4159 | 1 | 0.519 |
